# Letter from Dr. Perry; Gastro-Intestinal Catarrh

**Published:** 1890-02

**Authors:** J. F. Perry

**Affiliations:** Red Rock, Texas


### GASTRO-INTESTINAL CATARRH.—DR. WILSON'S CASES.

RED ROCK, TEXAS, January 23, 1890.

*Editor Daniel's Texas Medical Journal:*

While I deeply and profoundly sympathise with our professional brother, Dr. Wilson, in his great bereavement, permit me to make a few suggestions that may prove of benefit to our profession. Not that I deem myself capable of instructing a large number of our brethren, but my ideas may, perhaps, be in accord with some, and may, perhaps, be of advantage to others.

In gastro-intestinal catarrh, or the summer complaint of children, I have had quite a large experience, and in so far as the symptoms and general course of the disease runs, they are all pretty much alike, therefore a discussion of these is unnecessary, and I will merely give such treatment as I have found to be the most successful; I have usually adopted this course:

When the patient is first seen, and that is generally after the case has progressed several days, or, in some cases, weeks, I commence the treatment with a full dose of calomel, in order to unload the bowels of any hardened feces that may be there, and to arouse the portal circulation; if this should be accomplished, I then prescribe the following formula:

| | | |
|---|---|---|
| ℞ | Creta Prep. | 2 ʒ |
| | Aqua Pura | 6 ℥ |
| | Tr. Opii. | 80 gtt |
| | Camp. Tr. Opii. | 120 gtt |
| | Oil Sassafras | 60 gtt |
| | Tr. Camphor | 120 gtt |
| | Acitatis Plumbi | 20 grs |

Which I call, for want of a better name, chalk mixture. I order it given, if the case is severe, from one-third to one teaspoonful every two hours, if not so severe, the same dose after every operation, followed at night with a dose, according to age, of hydrarg. cum creta. The little sufferer is to be well nourished, and to have incorporated in the aliment a small dose of pepsin.

I agree with you that there is no specific; but the above course has given me better results and more satisfaction than any other I have tried. I only use quinine after the disease has been subdued. I then give it together with syrup of iodide ferri.

But, Mr. Editor, it often happens that, with this, as with all other remedies, we are doomed to disappointment. We often see our little patient gradually sink, until death closes the scene. All we can then do is to look up to Him who orders all things right, and submit to the divine behest with becoming Christian fortitude, feeling assured that if we prove but a tithe as gifted as they, we will one day see them again. Yours, etc.

J. F. PERRY.

